# Review of the Gate Structure for Normally Off p-GaN High-Electron-Mobility Transistors Towards High Performances

**DOI:** 10.3390/ma19112205

**Published:** 2026-05-23

**Authors:** Taofei Pu, Xiaobo Li, Liuan Li, Jin-Ping Ao

**Affiliations:** 1 School of Automation and Electrical Engineering, Shenyang Ligong University, Shenyang 110159, China; fbc_ptf@126.com; 2 Engineering Research Center of Internet of Things Technology Applications, Jiangnan University, Wuxi 214122, China; lixiaobo166@163.com (X.L.); jpao1800@jiangnan.edu.cn (J.-P.A.); 3 State Key Laboratory of Superhard Material, Jilin University, Changchun 130012, China

**Keywords:** gallium nitride, normally off operation, p-GaN layer, high-electron-mobility transistor, gate structure

## Abstract

As a representative wide-bandgap semiconductor material, gallium nitride (GaN) has attracted increasing attention because of its superior material properties (e.g., high electron mobility, high electron saturation velocity, and critical electric field). For power electronics applications, and to take full advantage of the superiorities of the GaN material, the normally off operation is required based on an AlGaN/GaN heterostructure. For a commercial approach, GaN HEMTs with a p-GaN gate have become a research hotspot. The characteristics of p-GaN gate HEMTs have a significant relationship with gate structure, especially the contact type on the p-GaN layer. In this review, the necessity of normally off operation and the advantages of adopting a p-GaN gate are elaborated, followed by the theory of achieving normally off operation by p-GaN and critical fabrication processes. The various gate structures are discussed, including metal gate, junction gate and hybrid gate structures on the p-GaN layer, to improve threshold voltage. Meanwhile, the methods required to optimize breakdown voltage and monolithically integrated technologies are also demonstrated. This review outlines the development and future trends of p-GaN gate HEMTs for power systems.

## 1. Introduction

Now, because of the increasing requirement of electric energy, the market scale of associated power electronics and semiconductors has reached US$100 billion globally. Further, it is expected to rise by over 100% by 2030 due to rapidly growing applications in electricity grids, renewable energy integration, electric vehicles (EVs), data centers and mobile electronics [1,2]. However, the most widely used silicon (Si)-based devices have reached the material theoretical limit and will not be able to adapt to the tremendous energy demand over the coming years. With decades of development, wide-bandgap (WBG) semiconductor devices, including silicon carbide (SiC), gallium nitride (GaN), gallium oxide (Ga_2_O_3_) and diamond, have demonstrated significant improvements in performance and efficiency, surpassing Si-based devices [3]. Among them, the SiC and GaN devices are the most representative and have been successfully commercialized. Due to their much wider bandgap, SiC and GaN have a Baliga’s figure of merit (BFOM) two orders of magnitude higher than Si and possess outstanding thermal stability simultaneously [4,5], as shown in Table 1. In combination with superior material properties and novel device structures, WBG devices have lower energy losses, higher frequency, and higher operating temperatures than Si devices [6].

Until now, SiC devices have demonstrated excellent performance in the field of high voltage, covering several voltage classes, namely 650, 900, 1000, 1200 and 1700 V, and low and medium switching frequencies. Meanwhile, GaN devices focus on low- and medium- voltage ranges (from 15 to 1200 V) and medium and high switching frequencies [7,8]. The GaN high-electron-mobility transistor, covering a voltage class of 650 V, is the most typical GaN device transistor (HEMT) in consumer electronics, being widely utilized as an important component in fast-charge applications.

## 2. The Realization of Normally Off Operation for GaN HEMTs

### 2.1. The Device Structure for Normally Off Operation

As a very cost-effective solution, GaN-on-Si lateral HEMTs (AlGaN/GaN hetero-structure HEMTs) have been successfully applied in high-efficiency compact-size converters. Their excellent performance including low on-resistance (*R_on_*), high breakdown voltage (*V_BD_*) and high switching frequency originates from the two-dimensional electron gas (2DEG). As the unique structure of GaN HEMTs, the 2DEG forms at the AlGaN/GaN interface, featuring a high electron mobility of around 1500 cm^2^ V^−1^ s^−1^ and an electron concentration of 1 × 10^13^ cm^−2^. Over the past decade, rapid advances have been achieved by industrial enterprises and academic institute progress in device design, epitaxial growth, fabricate processing, packaging technology, reliability improvement and gate driving techniques. GaN-on-Si lateral HEMTs have taken the lead in commercialization, emerging as the first batch of mainstream products ahead of SiC-based devices after a decade of development [9,10].

**Figure 1:**
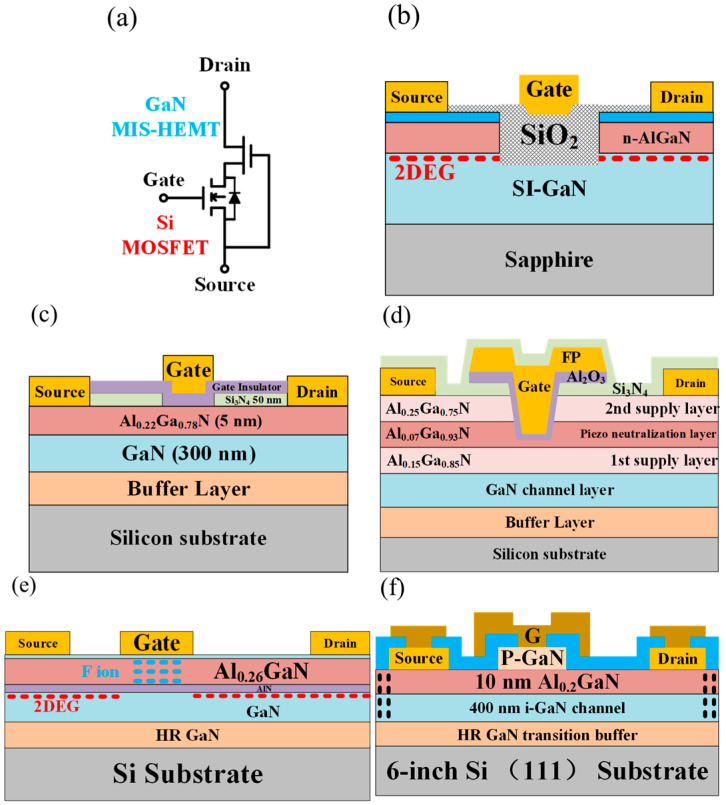
The methods of achieving normally off operation: (**a**) cascode [14], (**b**) recessed gate [15], (**c**) thin barrier layer [16], (**d**) piezo neutralization layer [18], (**e**) fluorine ion [20], (**f**) p-type cap layer [24].

However, AlGaN/GaN HEMTs suffer from a notable drawback in practical application in that a 2DEG channel operates in a normally on mode (depletion mode) at a gate voltage of 0 V, requiring an additional negative gate bias to deplete the channel. Then, a threshold voltage (*V_th_*) below 0 V inevitably induces a more complex circuit design, extra power consumption and potential safety hazards. Therefore, the normally off operation (enhancement mode) with *V_th_* > 0 V is required with high performance [11]. Moreover, a *V_th_* exceeding 3.0 V is expected to avoid noise-induced malfunction and realizing fail-safe operation [12,13]. Until now, numerous approaches have been developed to achieve normally off operation, including cascode (Figure 1a) [14], recessed gate (Figure 1b) [15], thin barrier layer (Figure 1c) [16,17], the introduction of a piezo neutralization layer (Figure 1d) [18], nonpolar a-plane channel construction [19], fluorine ion implantation (Figure 1e) [20,21], and p-type cap layer (Figure 1f) [22,23,24,25], among others.

The first technical route is to integrate additional normally off devices to form the cascode configuration (Figure 1a), which consists of a normally off Si-based metal–oxide semiconductor field-effect transistor (MOSFET) and a normally on AlGaN/GaN HEMT [26]. This dual-chip hybrid structure leverages the Si-based MOSFET architecture to achieve a low gate leakage current and elevates *V_th_*. However, this solution will introduce two main drawbacks. Firstly, there is a significant voltage fluctuation in GaN devices during high-frequency switching, and the parasitic inductance introduced by the bonding wire will induce extra gate voltage and further cause fault turn-on. Meanwhile, the parasitic inductance prolongs the switching time, leading to increased switching losses and degrading system efficiency. Secondly, Si-based devices generate high concentrations of carriers during high-temperature operation, causing device performance to deviate from design values. The second technical route is to reduce the concentration of 2DEG below the gate region (Figure 1b–d), which can achieve the synergistic optimization of *V_th_* and forward *R_on_*. Specially, the AlGaN barrier layer beneath the gate can be etched and form the recessed gate (Figure 1b), which has a capacity of obtaining a higher *V_th_*. Then, a gate dielectric layer is heteroepitaxial grown to suppress the leakage current due to the thin barrier layer. However, an inductively coupled plasma (ICP) dry etching is used for the fabrication of the recessed-gate structure, and the crystal damage and interface defect induced inevitably by ICP could seriously influence the device *V_th_* value and performance stability [27]. Although digital etching can mitigate a great amount of damage via cyclic oxidation and wet etching, it is difficult to ensure uniformity in the mass production [28]. Meanwhile, the interceptive 2DEG channel would lead to a slightly larger *R_on_*. In another approach, reducing the thickness or low Al content of the AlGaN barrier layer weakens spontaneous and piezoelectric polarization effects, resulting in insufficient electron density in forming a 2DEG channel (Figure 1c), which can form a natural normally off device. With respect to the recessed-gate scheme, this technique can offer a recess-free process and avoid dry etching damage. Meanwhile, the high density of 2DEG in the access regions can be obtained by depositing the insulator layer on top of the thin AlGaN barrier. However, the device performance is susceptible to the quality of the dielectric layer and the interface states between the insulator and barrier layer. Both the piezo neutralization layer and nonpolar a-plane channel demand stringent epitaxy growth conditions, which are difficult to achieve in a good performance device in normally off operation (Figure 1d).

**Figure 2:**
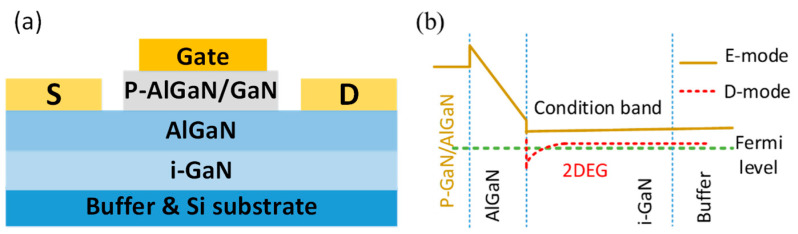
(**a**) AlGaN/GaN heterostructure HEMT with p-AlGaN/GaN cap layer; (**b**) energy band diagram of HEMT.

The third technical route is to introduce an additional charge to deplete the 2DEG below the gate electrode to achieve normally off operation. For the fluoride (F) ion implantation method (Figure 1e), the F-ions could deplete the 2DEG channel via strong electronegativity, thereby achieving a positive *V_th_*. The parameter instability under high electric stress and severe reliability problems need to be addressed [29,30,31]. Different than other techniques, the p-type cap layer is a promising method that requires neither recess etching nor the ion implantation process (Figure 1f), and the epitaxial growth is relatively straightforward. As illustrated in Figure 2a, this epitaxy structure is based on the AGaN/GaN heterostructure, where a p-type GaN is grown above the AlGaN barrier layer to realize normally off operation. The p-GaN is commonly doped with magnesium (Mg) compound Cp_2_Mg as precursors via metal–organic chemical vapor deposition (MOCVD) around 800–1000 °C. In detail, highly pure ammonia (NH_3_) and high-quality trimethyl gallium (TMG) serve as precursor chemicals, and Cp_2_Mg is used as the dopant source. The resulting 2DEG channel between the AlGaN/GaN and p-GaN layer at the gate region can be treated as a pin-diode structure with a depletion region. With proper doping concentration and thickness of p-GaN layer, the depletion region would surpass the AlGaN barrier layer and expand into the GaN layer at a 0 V bias, thus discontinuing the 2DEG channel at the gate region. This transformation shifts the device operation from normally on to normally off mode. The energy band diagram with the p-GaN layer is presented in Figure 2b. With respect to the normally on AlGaN/GaN HEMT structure, the conduction band of the channel region is lifted above the Fermi level, indicating a negligible electron density in the channel at a *V_gs_* of 0 V. Yet, with positive gate bias, the 2DEG channel is rebuilder-established, enabling the electric condition between the source and drain electrodes [32].

### 2.2. The Epitaxy Structure and the Preparation of the p-GaN Gate

**Figure 3:**
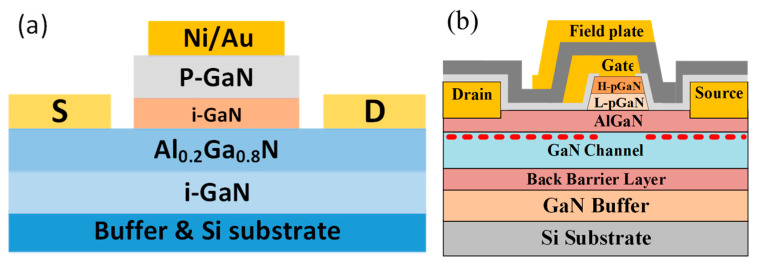
(**a**) Cross-section schematic view of the p-GaN HEMT with the i-GaN blocking layer [22] and (**b**) engineered p-GaN doping profile [34].

Due to the high ambient temperature of p-GaN growth in MOCVD, the element diffusion is inevitable. If the Mg diffusion region extends to the 2DEG channel, the *V_th_* increases owing to a lower electron concentration. However, the sheet resistance would increase dramatically, resulting in a critical trade-off between *R_on_* and *V_th_*. For decreasing the influence of Mg diffusion to a device, researchers have explored various strategies, including optimizing the growth conditions, altering the Mg activation temperature or activation sequence (after the process), and changing the process parameters. These approaches enable a positive shifting *V_th_* while maintaining a similar Mg doping concentration [33]. In addition to optimizing the epitaxy and process conditions, an intrinsic GaN is inserted between p-GaN and AlGaN to act as a blocking layer to suppress the Mg diffusion (Figure 3a) [22]. According to experimental results, the 2DEG channel performance of the device incorporating a 20 nm i-GaN blocking layer is close to the reference ones, indicating that the i-GaN is effective in hindering Mg diffusion. However, the *V_th_* shifts to around 0 V, because the blocking layer will weaken the control capability of p-GaN over the channel. Therefore, a 10 nm blocking layer is selected as a compromise to balance channel resistance and *V_th_*. In addition, an engineered Mg doping profile in the p-GaN layer is also proposed for improving further device performance in Figure 3b [34]. Owing to the low-doping p-GaN/AlGaN heterostructure, a low gate capacitance together with an excellent gate-to-channel modulation ability are obtained. In contrast, a p-GaN layer with a high doping concentration is beneficial for improving gate depletion capability and reducing gate contact resistance. Finally, a power-added efficiency (PAE) of 70% is achieved together with an output power density of 1 W/mm at a *V_DS_* of 10 V. Generally, due to the high activation energy of Mg (170–250 meV), the Mg activation efficiency is only about 1–3% even at a high Mg doping concentration of 10^20^ cm^−3^ [12,35]. And due to the passivation effects of hydrogen (H) atoms and self-compensation, the hole concentration is further limited [36,37]. Consequently, the *V_th_* of p-GaN-gated HEMTs is commonly around 1 V, which is insufficient in practical applications to ensure safe operation. The *V_th_* of the GaN HEMT with p-GaN can be determined by the p-GaN doping concentration, the thickness and the Al component of the AlGaN barrier layer. This is an inherent trade-off between the 2DEG concentration and *V_th_*, e.g., commonly, a low 2DEG concentration generally means a high *V_th_*, and vice versa.

**Figure 4:**
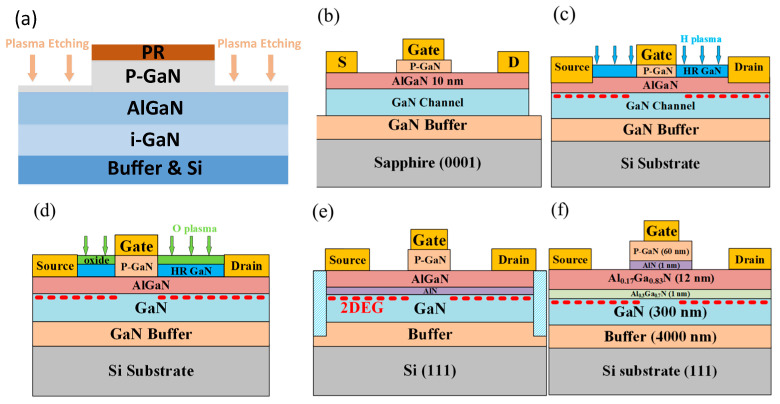
(**a**) p-GaN dry etching for constructing the gate stacks, (**b**) selective-area growth of p-GaN [40], (**c**) hydrogen plasma treatment [41], (**d**) oxygen plasma treatment [42], and (**e**) self-terminated p-GaN removal process with Cl_2_/N_2_/O_2_-based gas [43] and (**f**) F-based gas [45].

In a normally off GaN HEMT with a p-GaN layer, the p-GaN layer in the access region is removed to recover the 2DEG channel, as shown in Figure 4a. The common dry etching process with chlorine (Cl_2_) or boron trichloride (BCl_3_) plasma has high etch rate but is prone to causing under-etching or over-etching of the p-GaN grown on the AlGaN/GaN at the non-gated access region [38]. In addition to the issue of precise etching thickness control, a plasma/ionic bombardment and etching defects or damage would reduce the 2DEG density and deteriorate device performance [39]. For alleviating the etching issue, a low-power etching recipe and precise control of the etching depth are commonly carried out to maintain a high electron density in the access region. Alternatively, the selective-area growth of p-GaN, an approach that does not involve the etching, has been adopted, as shown in Figure 4b [40]. Nevertheless, the selective-area growth process is quite complex and has a high cost; thus, it is used less. In addition, a p-GaN passivation approach using hydrogen (H) plasma was proposed. In this scheme, the acceptors and H form a Mg-H neutral bond, thereby compensating holes in the p-GaN [41]. Then, the conductive p-GaN in the access region is converted into a high-resistance (HR) state while the 2DEG beneath can be restored, as depicted in Figure 4c. Like the H plasma treatment, the oxygen plasma treatment has a similar passivation effect as that shown in Figure 4d, which can form Mg-O bonds [42]. Recently, the much more popular approach is the self-terminated p-GaN removing process, which uses a Cl_2_/BCl_3_/CHF_3_ or Cl_2_/N_2_/O_2_-based mixture as the etching gas [43,44]. In Figure 4e, with a conventional epitaxy structure, the Al atoms with Ga atoms nearby form a network-like (Al,Ga)O_x_ thin film with a high bond energy when the O-containing ICP etching reaches the AlGaN barrier, acting as an etching-resistant oxide layer. With respect to Cl_2_/N_2_/O_2_-based gas, a BCl_3_ + CF_4_ etching gas mixture is also utilized, paired with an inserted epitaxy AlN layer in Figure 4f. The AlN interlayer is positioned between the p-GaN and AlGaN barrier layer, which can react with CF_4_ to form nonvolatile aluminum fluoride (AlF_3_) that can terminate the etching process precisely at the AlN layer [45,46].

Alternatively, other p-type materials, such as NiO [47], CuO/Cu_2_O [48], and SnO [49,50], as well as p-type semiconductors including p-AlGaN and p-GaN, are also employed to construct the gate structure. Although all of these p-type materials can lift the heterostructure energy band, only p-AlGaN/GaN can lead to a full depletion of the 2DEG at a gate bias of 0 V, as shown in Figure 2a,b. With respect to p-AlGaN, the p-GaN is more favorable for epitaxy growth and widely utilized due to its relatively lower activation energy.

## 3. Advanced p-GaN Gate Configurations Towards High Performances

For improving the performance of p-GaN gate HEMTs, especially in terms of *V_th_* and gate leakage current, various optimization strategies have been proposed. The gate types can be classified into metal gate (ohmic, Schottky on p-GaN), junction gate (MIS, PN gate on p-GaN), and hybrid gate. At present, the metal gate is widely applied in most commercial normally off GaN devices in practice. And other gate architectures are still under ongoing development.

### 3.1. The Commonly Used p-GaN Gate with Metal Electrodes

**Figure 5:**
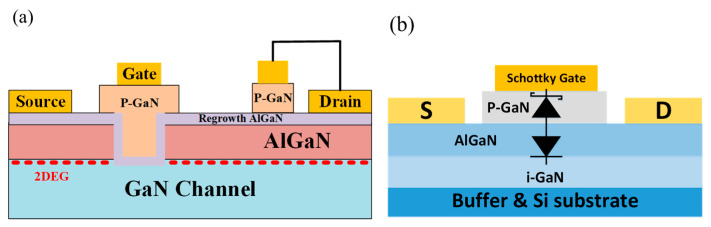
(**a**) Proposed TRRG HD-GIT device [52]; (**b**) schematic of the back-to-back diode model for Schottky gate device.

The first type of metal gate electrode on p-GaN is the ohmic gate. Metals with a high work function, such as Ni, palladium (Pd) and NiO/Ni stacks, could form ohmic/ohmic-like contacts with the p-GaN/AlGaN structure after annealing at around 500 °C in an O^2^-containing ambient environment. Due to the hole injection phenomenon at a high *V_g_* (>*V_f_*, pn junction built-in voltage), the ohmic gate is also referred to as a gate-injection transistor (GIT). A distinctive characteristic of the ohmic gate is its transconductance curve featuring two peaks [51]. Based on the ohmic gate, a hybrid drain embedded GIT (HD-GIT) and “through recess and regrowth gate (TRRG) technology” are proposed in Figure 5a [52], which utilize the hole injection at the recess region and the regrowth process to mitigate current collapse and *V_th_* non-uniformity issues. The ohmic gate operates on a principle similar to that of a JFET. However, due to a turning-on voltage of around 4 V for the PN junction (gate-to-source) and a consequent large gate leakage current, the gate swing needs to be limited, while complex gate driving schemes are required for monolithically integrated circuits. As a current-driving device, the ohmic gate can offer great *V_th_* stability and good dynamic on-resistance (*R_on_*) [53,54]. And the low *V_th_* value is insufficient for practical application, prompting the development of novel device construction.

For further improving *V_th_* and suppressing the gate leakage current, a gate metal electrode with Schottky contact on a p-GaN layer has been proposed. Such a Schottky gate is commonly achieved by using a low-work-function metal or alloy, such as Ni, Ti, TiN, Al, V, W, or Mo, in contact with p-GaN. The theoretical model of the Schottky gate is shown in Figure 5b. In essence, this stack is electrically equivalent to a back-to-back diode model: a metal/p-GaN Schottky diode combined with a p-GaN/AlGaN/GaN p-i-n diode [55]. For the upper diode in Figure 5b, the Schottky junction is reversed-biased when forward gate bias is applied. Then, the hole depletion layer on the p-GaN surface can withstand a portion of the gate bias, thereby suppressing the gate leakage current as well. And the Schottky barrier height *Φ_B_* and depletion layer width increases as the gate metal work function decreases. Thus, a higher *V_th_* in the p-GaN gate HEMT can be obtained using a lower gate work function [12,56]. And the gate swing is also enlarged to above 7 V [57,58].

With respect to the ohmic gate, the Schottky gate can offer a higher *V_th_* and a lower gate leakage current, which arises from the depletion layer at the surface of the p-GaN [59,60]. Meanwhile, drawbacks of the Schottky gate also exist. First, due to its location between the Schottky junction and the AlGaN/GaN heterojunction, the electric potential of the p-GaN layer is in a state of “floating” without any directional connection to metal termination. Second, the *V_th_* instability emerges under positive gate bias stress, which refers to different mechanisms according to the value of the gate bias [61,62,63]. In addition, the Schottky interface state, like dangling bonds and defects caused by ICP, severely degrades device performance, making the surface preparation or passivation of p-GaN indispensable [64]. In contrast, although the ohmic gate suffers from a relatively low *V_th_* and a large gate leakage current, it has a more stable device performance. Compared to a current-driving device for the ohmic gate, the Schottky gate is a voltage-driving device. Thus, the gate-driving circuit design in the application needs to be taken into account.

**Figure 6:**
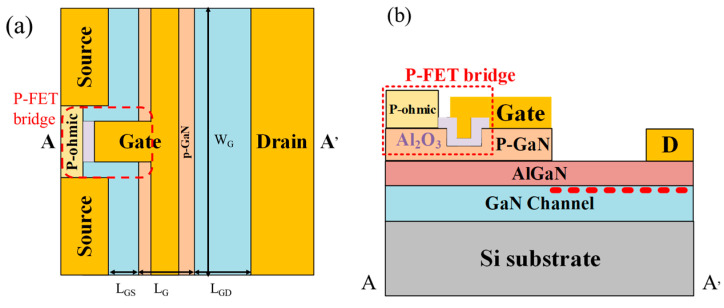
(**a**) Top view of the p-GaN gate HEMT with the p-channel FET bridge (W_p_/W_G_ = 1/4); (**b**) 3D schematic cross-sectional view along the A-A’ cutline [65].

A novel gate structure is proposed to improve *V_th_* and *V_th_* stability by using a normally on p-channel FET bridge connecting the source and Schottky gate, as shown in Figure 6 [65]. The mechanism of a high *V_th_* is mainly ascribed to the drastic voltage drop on the Schottky junction owing to the p-channel turning off when the *V_gs_* exceeds the *V_th_* of the p-FET (*V_th,pFET_*). Hence, the *V_th_* can be modulated within a wide range from 3.6 to 8.2 V by controlling the p-FET gate recess depth range. And due to the normally on performance of the p-FET, the “floating p-GaN layer” is eliminated by connecting the source via the p-FET channel. Thus, the merits of the Schottky gate, including a low gate leakage current and a large gate swing, can be retained, and the stable *V_th_* can be obtained. The innovative gate topology structure is implemented to mitigate many drawbacks that are challenging to address in a conventional structure. This trend of transitioning from discrete devices to monolithic integration is expected to become a research hotspot for GaN-powered devices.

### 3.2. The Junction Gate Structure for p-GaN HEMTs

**Figure 7:**
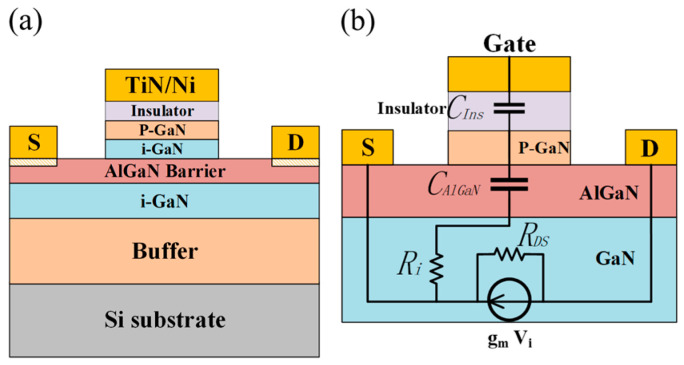
(**a**) Schematic of the MIS gate device structure [71]; (**b**) equivalent circuit model of MIS gate device [13].

The practical utilization of the common metal gate is still limited owing to the low turn-on voltage of the p-GaN/AlGaN/GaN junction and the maximum allowable *V_GS,max_* (<7 V). Meanwhile, a *V_GS_* of 5 V is required to fully turn on the 2DEG channel to achieve a low *R_ON_*, leaving a narrow safety margin between the operating *V_GS_* and *V_GS,max_* [66,67,68]. And, for mitigating the poor anti-noise issue, a *V_th_* above 3 V during application is required [69]. Furthermore, other issues are the low gate swing and large forward gate leakage current led by the turning on of the p-n junction formed by the p-GaN gate and 2DEG, which would result in power loss and lowering the peak value of the current density [70]. For mitigating metal gate issues, some researchers have used a junction structure on p-GaN, including a metal-insulator–semiconductor (MIS) structure and a PN junction, as shown in Figure 7a [13,71,72].

With respect to the ohmic gate, the MIS gate can be regarded as a gate with serial circuit consisting of insulator capacitance and AlGaN capacitance (unique capacitance in an ohmic gate device), as shown in Figure 7b. When a gate voltage (equal to the *V_th_* of an MIS gate) is applied to the MIS p-GaN gate HEMT, the insulator capacitance is charged first, and then the AlGaN capacitance. Namely, the insulator layer undertakes a portion of the gate voltage, while the remaining gate voltage (equal to *V_th_* of ohmic gate) is used to restore the 2DEG channel. According to the experiment, both the *V_th_* and gate breakdown voltage (*V_GBD_*) exhibit a positive linear correlation with SiN_x_ thickness, which confirms that the MIS gate structure could improve gate performance. In addition, in Formula (1), the relationship between the *V_th_* (MIS) and total capacitance of the MIS gate HEMTs is shown, where *V_th_* (ohmic) is the *V_th_* of the ohmic gate, *V_th_* (MIS) is the *V_th_* of the MIS gate, and *C_Ins_* and *C_AlGaN_* are the insulator and AlGaN capacitance, respectively. In this formula, it can be deduced that a lower *C_Ins_*, namely, a thicker insulator layer, would lead to a higher *V_th_* (MIS). Based on this theory, a high *V_th_* (MIS) of about 6 V and a large *V_GBD_* of 26 V is achieved with 26 nm SiN_x_, and there is a much lower gate leakage current [70].

(1) $$C_{AlGaN}\cdot V_{th}\left(ohmic\right)=C_{Ins}\cdot \left\{V_{th}\left(MIS\right)-V_{th}\left(ohmic\right)\right\}\;V_{th}\left(MIS\right)=\frac{C_{AlGaN}+C_{Ins}}{C_{Ins}}\cdot V_{th}\left(ohmic\right)$$

**Figure 8:**
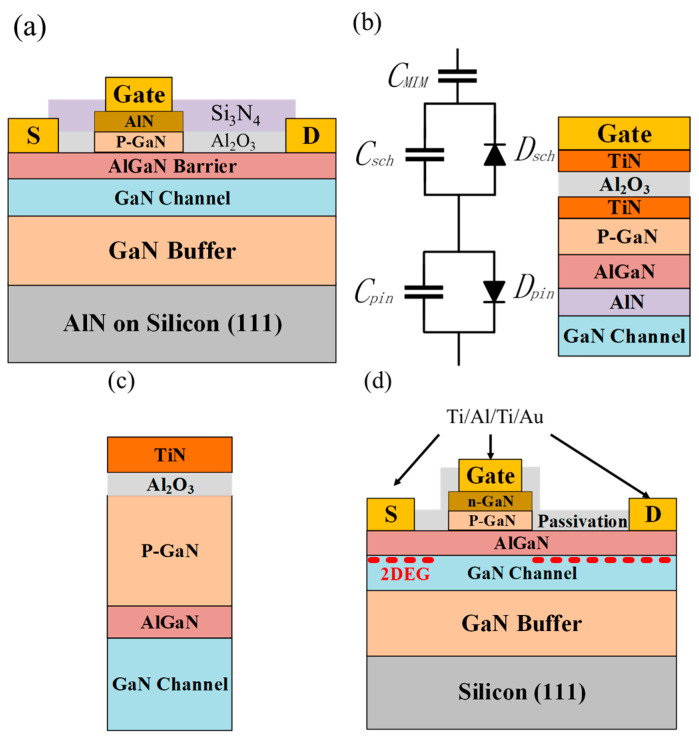
(**a**) Cross-sectional structures of AlN/p-GaN gate HEMT, equivalent circuit model of p-GaN gate HEMT combining ultrathin Al_2_O_3_ layer [73] with (**b**) MIM [75] and (**c**) MIS gate stacks [76], and (**d**) PNJ gate structure without insulator deposition [72].

However, a thicker insulator layer commonly results in a low transconductance value, which is ascribed to the increased distance between the gate electrode and the 2DEG channel, as well as the enlarged gate length. Meanwhile, interface traps exist at the insulator–p-GaN interface, which degrade device reliability and stability. To address these issues, thinner insulators with a high-k are adopted, which could shrink the gate thickness and improve the gate controlling the channel. Numerous researchers selected AlN, as shown in Figure 8a, either grown in situ or by atomic layer deposition (ALD), as the gate insulator in contact with the p-GaN layer [73,74]. There has been significant progress in *V_th_*, gate swing, *V_BD_* and lifetime. Further, for mitigating the drawback of the “floating p-GaN layer” in the MIS structure, an ultrathin Al_2_O_3_ film deposited by ALD as the insulator layer is purposed to form the tunneling effect without carrier trapping in Figure 8b,c. As the Al_2_O_3_ thickness decreases to 3 nm, carriers mainly pass through the Al_2_O_3_ barrier via direct tunneling, avoiding capture by the interface and bulk defects, which have been deeply investigated by [75,76,77,78,79]. This is the essential difference between the MIS tunneling structure and the traditional MIS structure. These studies not only mitigated the drawbacks of the conventional MIS gate but also strengthened the device static performance, especially for dynamic stability. This demonstrates that the method of the MIS gate HEMT approach also exhibits a high-reliability performance. In addition to the MIS gate structure, a specialized gate structure using a PN junction (PNJ) has been demonstrated, as shown in Figure 8d. Similar to the reverse Schottky junction, the reverse-biased PNJ can undertake a higher voltage, resulting in a positive shift *V_th_*, a larger gate swing and a higher gate *V_BD_* than the Schottky gate. Moreover, due to its simple fabrication process (without insulator deposition) and the ohmic contact with the gate electrode, the PNJ gate structure can lend more stable and reliable device characteristics [72].

For pursuing further optimized device parameters, a junction that can undertake a higher voltage than the Schottky junction is required. Therefore, the MIS structure as a radical solution is proposed, which could increase the *V_th_* and drastically lower the gate leakage current by increasing the thickness of the insulator. However, similar to the Schottky gate, the issue of the “floating” p-GaN layer always influences the gate stability. Then, the utilization of an ultrathin insulator with a high-k allows the occurrence of electron tunneling that promotes the stability of the MIS structure, simultaneously showing excellent device parameters with respect to that of the conventional MIS structure. As a consequence, the solution of the ultrathin insulator paves the way for practical application.

### 3.3. The Hybrid Structure by Combining Metal and Junction Gates

**Figure 9:**
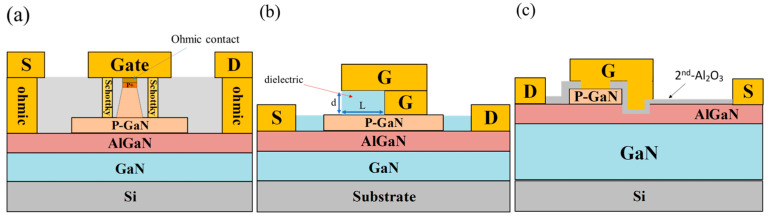
Cross-sectional structures of (**a**) Schottky–ohmic hybrid gate [80], (**b**) Schottky–MIS hybrid gate [83], and (**c**) Schottky recessed–MIS hybrid gate [84].

Apart from the aforementioned single-structured gate configurations, a hybrid gate integrating two different gate architectures has been proposed to optimize parameters and offset the inherent limitations of a single-structure gate. Among such designs, hybrid-gate structures primarily combining Schottky gates with an ohmic gate or a Schottky gate with an MIS gate have attracted extensive attention. As illustrated in Figure 9a, the Schottky– ohmic hybrid gate incorporates the merits of both gate types [80,81]. The ohmic-type p-GaN region forms a free-carrier “discharge path”, which suppresses the “floating” gate effect and thereby enhances the *V_th_* stability. And the peripheral geometry design of the Schottky gate can take full advantage of the depletion region, ensuring a relatively low gate leakage current. Moreover, the influence of the relative positional relationship and length ratio between the ohmic and Schottky gate segments on characteristics is also investigated in [82]. The optimal Schottky–ohmic hybrid gate can improve the maximum drain current with compromising a slight rise in the gate leakage current. As shown in Figure 9b, the Schottky–MIS hybrid gate HEMT features a higher *V_th_* and a lower forward gate leakage current than that of the conventional Schottky gate p-GaN HEMT, and device performance can be effectively modulated by parameters of the gate dielectric layer according to simulation results [83]. Meanwhile, the hybrid gate integrating the normally off Schottky p-GaN gate and normally on MIS gate could also improve the device stability, as shown in Figure 9c. The drain-induced dynamic *V_th_* shift can be reduced owing to the normally on MIS gate shielding the interplay between the drain and the p-GaN region, and a decent saturation current and on-resistance are exhibited [84]. Moreover, the drain-side MIS gate can be regarded as a junction termination, which could transfer the electric field peak from the Schottky p-GaN gate to the MIS gate; this contributes to better reliability [85]. In such hybrid gate architectures, the Schottky gate section shows the advantage of a high *V_th_* and low gate leakage current, and the ohmic gate section facilitates carrier transport and alleviates electric potential isolation effects in the p-GaN region. Due to having no directional connection to the metal termination, the normally on MIS gate section is used to improve device stability and lower electric field crowding near the p-GaN to the drain region. In a word, the hybrid gate is a cost-effective method that can be used to pursue better device parameters, which comprises the existing gate structure. With respect to devising a novel gate structure, this method is relatively simple and more feasible.

The key parameters of devices equipped with diverse p-GaN gate structures are summarized in Table 2 for comparison. Among the metal electrode gates, the Schottky gate exhibits overall superior performance compared with the ohmic gate. Their gate leakage current remains extremely low even under elevated gate voltages, which translates to lower power loss at application. This further verifies that the Schottky gate possesses a higher *V_th_* and other comparable parameters, making it more suitable for industrial practical applications. Admittedly, adopting the regrowth technique and sophisticated processes can also optimize the ohmic gate parameters; yet, the associated manufacturing costs cannot be neglected. In addition, to adopt the pFET–bridge structure, the device could obtain a higher *V_th_* by modulating the turn-on voltage of the integrated pFET, which points out a potential direction for future device development. 

For junction gates, their core advantages lie in a high *V_th_*, a large gate voltage swing and a low gate leakage current, all of which are essential for practical engineering applications. However, they inevitably suffer from a slightly higher *R_on_* and a more serious electrical isolation of p-GaN compared with the Schottky gate. Furthermore, a junction gate embedded with an MIM structure adopts an ultrathin insulator layer to realize the tunneling effect, effectively alleviating the “floating” gate issue. Nevertheless, there is a trade-off between the gate voltage swing and leakage current. As a novel method, hybrid gates serve as a balanced compromise among the above schemes. Though they integrate the features and performance of both gate types, they increase fabrication complexity and remain in the research and development stage. Currently, metal electrode gates, especially Schottky gates, still dominate the mainstream consumer market. Junction gates and hybrid gates are merely limited to academic discourse; however, they are the potential development direction and are expected to deliver better device performance in future.

## 4. The Further Optimization of Device Structure for High Performances

In response to the development trend of high-power and high-frequency applications in the future, p-GaN devices still need to make breakthroughs in the following two critical aspects. Firstly, in order to improve their processing power density, the devices must be able to withstand higher operating voltages, i.e., higher device breakdown voltage. Secondly, in order to adapt to high-frequency operation, it is necessary to further integrate p-GaN power devices with other peripheral circuits on a single chip to reduce parasitic capacitance and inductance.

### 4.1. The p-GaN-Based Termination for Breakdown Voltage

Now, various types of GaN devices have been commercialized. According to the voltage ranges, the devices are mainly classified into low voltage (*V_DS,max_* < 200 V), middle voltage (*V_DS,max_* from 200 to 650 V) and high voltage (*V_DS,max_* up to 1 kV and above). Due to their low cost and high epitaxial quality, low- and middle-voltage GaN devices have been widespread adopted and are commonly utilized in the application of DC–DC converters, wireless microwave transfer, home appliances, telecommunication servers, data centers and servo motors [91,92,93,94,95]. However, the high-voltage requirement of electric vehicles and industrial motors is limited by the vertical breakdown characteristics of GaN on Si. There are two methods to enhance the device breakdown voltage (*V_BD_*): adjusting the epitaxy structure and introducing a gate termination.

**Figure 10:**
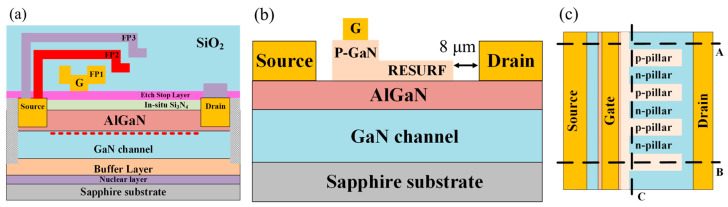
(**a**) The fabricated GaN HEMTs with in situ SiN_x_ and field plate design on a 6 in sapphire [97]; (**b**) schematic diagram of the RESURF HEMT [87]; and (**c**) the top view of the superjunction p-GaN gate HEMT (SJ-HEMT) [89].

As a mature substrate material in the field of optoelectronics, the sapphire substrate has attracted a lot of attention due to its natural insulating properties. Meanwhile, owing to reduced electric fields around the buffer layers, the thick buffer layer is not necessary in the insulating sapphire substrate [96]. Research on a 1200 V GaN transistor based on sapphire has been carried out. A normally on GaN HEMT with an off-state leakage current <2 μA at 1200 V and a catastrophic breakdown >2 kV was demonstrated in [96]. Moreover, collaborating with multilayer field plates, an in situ SiN_x_ passivation layer and a sufficient *L_gd_* of l00 μm, a *V_BD_* of >8 KV was also reached on a 6-inch sapphire substrate, as shown in Figure 10a [97]. With a undoped ultrathin buffer layer of 100 nm, a normally off 1200 V rated p-GaN gate HEMT was achieved, and the *V_BD_* reached 2300 V with an *L_gd_* of 22 μm in [90].

Based on the sapphire substrate, the gate termination extension could further enhance the device *V_BD_*, which originates from a thin p-GaN layer remaining after the p-GaN etching process, extending from the p-GaN gate towards near the drain. The remaining p-GaN could screen the surface traps and form a reduced-surface-field (RESURF) structure (Figure 10b). Thus, avoiding immaturity breakdown and effectively relaxing the electric field crowding at the gate edge to the drain can be achieved, leading to a higher *V_BD_* [86,87]. By this gate termination extension, normally off p-GaN gate HEMTs on a sapphire substrate demonstrated a high *V_BD_* of 2 kV and 10 kV corresponding to an *L_gd_* of 27 and 100 μm, respectively. Although, on the Si substrate this termination extension can also improve the *V_BD_* to 900 V [88]. In addition, a lateral superjunction (SJ) p-GaN gate HEMT on a sapphire substrate is proposed in Figure 10c, which can form the charge balance through the alternative p-/n-pillars that could obtain a more uniform electric field distribution. Based on this design, the SJ-HEMTs reveal a high *V_BD_* of 2655 V and an excellent dynamic performance [89].

In the high-voltage field, Si devices occupy an important position due to their mature industrial infrastructure. However, SiC has attracted widespread attention since its first application in electric vehicles. After decades of development, its market share in the high-voltage sector has been gradually expanding. As another wide-bandgap semiconductor, GaN exhibits material properties comparable to SiC and delivers significant superiority in switching frequency. Leveraging its advantage in switching frequency, GaN devices are expected to steadily enhance the conversion efficiency and power density of high-voltage systems while reducing the overall footprint and size. Therefore, improving the breakdown voltage of GaN devices is of great industrial importance.

### 4.2. The Logic Circuits for Monolithic Integration

Recently, GaN products have been gradually expanding from the consumer electronics sector to the industrial electronics field. With GaN technology demonstrating significant potential in single-stage onboard chargers (OBCs) and the rapid growth of AI power supplies, the GaN power device market is poised for explosive growth. The market size is projected to grow from approximately $600 million in 2025 to over $900 million, representing an increase of more than 50%. To address the increasing market demand and economic benefits, further leveraging the advantages of GaN materials, innovating device structures, and replacing existing power devices have become the directions for development. To fully unleash the potential of GaN power devices, it is necessary to monolithically integrate power switching devices with peripheral circuits such as controllers and drivers in an all-GaN configuration to minimize parasitic parameters.

**Figure 11:**
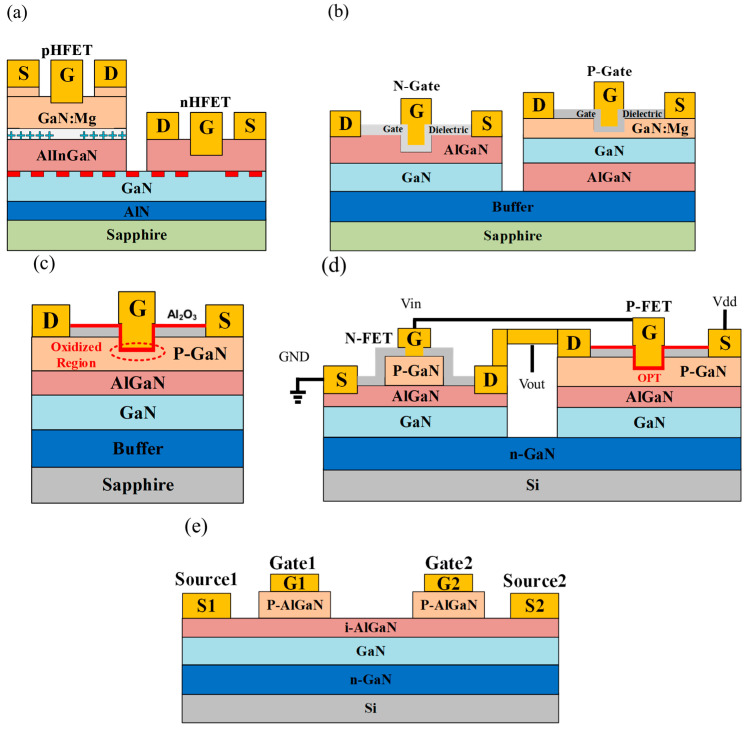
Schematic of (**a**) the first MI of GaN p/n-channel HFETs on a single wafer [98], (**b**) GaN NMOS and PMOS [99], (**c**) a normally off GaN PMOS using OPT technology [100], (**d**) a GaN CMOS inverter based on a commercial p-GaN gate power HEMT platform [101] and (**e**) a monolithic AlGaN/GaN bidirectional switch using double-gate structure [102].

Complementary logic (CL) circuits are one of the key elementary circuits enabling GaN monolithically integration. Compared with direct-coupled FET logic (DCFL), complementary metal–oxide semiconductors (CMOSs) could offer significant advantages in terms of power consumption, noise margin, drive capability, and design simplicity. The first MI of normally off p/n-channel transistors is achieved on a GaN/AlInGaN/GaN double heterostructure platform in Figure 11a [98]. The n/p-channel heterostructure field-effect transistors (HFETs) employ a 2DEG/2-dimensional hole gas (2DHG) to obtain inverting and rail-to-rail output characteristics. However, due to the absence of an MIS gate structure, the high gate current limits the saturation of logic “low” output voltage (*V_OL_*). The actual GaN CMOS is reported by using the selective-area epitaxy technology. The AlN/SiN dielectric stack is served as the gate oxide for NMOS and PMOS in Figure 11b [99]. Even if the device performance is lacking, the functional inverter IC confirms the feasibility of the GaN CMOS technology. Since the fabrication of the normally off p-channel device commonly requires a dry etching process to remove a portion of the p-GaN, defect repair becomes necessary. In Figure 11c, a p-channel normally off GaN metal–oxide semiconductor field-effect transistor (MOSFET) with a *Vth* of −1.7 V is achieved. This is realized by implementing an oxygen plasma treatment (OPT), which converts the top portion of the GaN layer to a hole-free state [100]. Utilizing this technology, GaN CMOS logic circuits were fabricated on a commercial p-GaN gate power HEMT platform, as shown in Figure 11d. The rail-to-rail operation and ultralow static power dissipation are confirmed. In addition, other logic gates, including not-and (NAND) gates, not-or (NOR) gates and transmission gates, are demonstrated as well [101].

The monolithic bidirectional switch (MBDS) based on the AlGaN/GaN heterostructure represents one of the most significant breakthroughs in recent years, having gained market recognition, particularly in inverter applications. As illustrated in Figure 11e, its double-gate structure enables bidirectional current conduction and voltage blocking in both polarities. These superior characteristics make it widely applicable in power converters, motor drives, and battery management systems (BMSs) [102]. Compared to the traditional BDS approach—which utilizes two discrete MOSFETs or insulated-gate bipolar transistors (IGBTs) in an anti-parallel or anti-series configuration—the MBDS achieves higher AC-AC conversion efficiency, faster switching speed, and a more compact system volume [103]. Monolithic integration stands as a key development trend of GaN technology, marking the evolution of GaN devices from discrete devices to integrated ones. Although overall it is still in the R&D stage, individual devices like MBDS have exhibited the trend of replacing Si-based circuits, which can provide superior conversion efficiency. Hence, this field has drawn tremendous attention from researchers worldwide.

## 5. Conclusions

As a mature large-scale industrial and commercial GaN device technology, p-GaN gate HEMTs have attracted significant attention and emerged as a research focus. This paper first elaborates on the superiority and operating principles of p-GaN gates to achieve normally off operation. Meanwhile, with regard to the p-GaN etching process, various critical fabrication methods are presented. Then, the evolution of p-GaN gate structures, based on the p-GaN/AlGaN/GaN epitaxial stack, to achieve higher threshold voltage, higher breakdown voltage, and more stable device characteristics, is summarized. In particular, the threshold voltage can be improved by some gate structures such as a Schottky metal gate, junction gate, and hybrid gate. And the breakdown voltage is enhanced by means of a thin buffer layer on a sapphire substrate and gate termination extension. To address the increasing market demand and economic benefits, the monolithic integration of GaN devices is also widely discussed to further leverage the advantages of the GaN material. This review offers a crucial outlook and development trends, thereby laying a solid foundation for the future design and performance enhancement of p-GaN gate HEMTs and related power electronic devices.

## Figures and Tables

**Table 1:** Comparison of material parameters of Si, GaAs, 4H-SiC, GaN, Ga_2_O_3_, and diamond.

Materials	*E_g_*	*ε*	*µ_n_*	*E_c_*	*V_sat_*	Thermal Conductivity	BFOM
Si	1.12	11.7	1450	0.3	1	130	1
GaAs	1.42	12.9	8500	0.4	2	54	15
4H-SiC	3.26	10	950	3.0	2	500	340
GaN	3.39	9	1000	3.3	2.5	130	870
β-Ga_2_O_3_	4.8–4.9	10	300	8	2	20	3444
Diamond	5.6	5.7	4000	10	3	2000	24,664

*E_g_* (eV), *ε*, *µ_n_* (cm^2^/V·s), *E_C_* (MV/cm), *V_sat_* (10^7^ cm/s) and BFOM (*ε_µ_E_b_^3^*, to Si) are energy bandgap, relative dielectric constant, electron mobility, critical electric field, saturation velocity and Baliga’s figure of merits, respectively. Thermal conductivity (W/m·K) [4,5].

**Table 2:** Summary of device performances with different p-GaN gate structures.

Gate Type	*V_th_* (V)	*V_GS,max_* (V)	Gate Leakage (mA/mm)	*R_on_* (mΩ·cm^2^)	*V_BD_* (V)	Reference
Metal electrode (ohmic)	1.2	/	≈10 (*V_g_* of 6 V)	523.74	670	[22]
-	1.1	/	<10^−2^ (*V_g_* of 6 V)	0.25	206	[34]
-	1.0	6	0.05 (*V_g_* of 6 V)	2.6	800	[51]
-	2.3	/	/	0.04	>980	[52]
-	1.0	/	/	2.6	640	[54]
-	0.9	/	≈0.1 (*V_g_* of 3.5 V)	6.42	2000	[86]
-	0.5	/	≈10 (*V_g_* of 5 V)	0.069	3400	[87]
-	1.4	/	≈0.8 (*V_g_* of 3.5 V)	5.4	1791	[88]
-	0.9	/	≈0.8 (*V_g_* of 3.5 V)	3.5	2655	[89]
Metal electrode (Schottky)	1.75	10.5	≈3 (*V_g_* of 8 V)	1.8	1100	[11]
-	3	15	0.02 (*V_g_* of 10 V)	3.36	/	[12]
-	2.5	/	/	0.8	600	[23]
-	1.6	/	≈0.8 (*V_g_* of 8 V)	3.92	1344	[24]
-	1.75	/	≈0.1 (*V_g_* of 6 V)	4.55	393	[41]
-	1.02	/	≈0.1 (*V_g_* of 8 V)	4	660	[42]
-	1.1	/	<10^−2^ (*V_g_* of 8 V)	1.6	300	[43]
-	1.7	/	10^−3^ (*V_g_* of 8 V)	0.75	256	[45]
-	1.1	/	10^−4^ (*V_g_* of 8 V)	0.045	/	[60]
-	1.82	12.1	≈0.1 (*V_g_* of 8 V)	2.5	/	[64]
-	1.9 (HV)	/	0.01(*V_g_* of 6 V)	0.004	2300	[90]
Metal electrode (pFET–bridge)	3.62	/	10^−3^ (*V_g_* of 10 V)	0.057	620	[65]
Junction gate (MIS)	2.8 (20 nm SiN_x_)	/	≈0.04 (*V_g_* of 15 V)	4	/	[13]
-	1.5	/	10^−8^ (*V_g_* of 5 V)	0.14	/	[69]
-	6 (20 nm SiN_x_)	26	10^−7^ (*V_g_* of 20 V)	216.2	/	[70]
-	2 (20 nm SiN_x_)	>15	10^−5^ (*V_g_* of 15 V)	141.3	670	[71]
-	3.9	17.6	/	6.31	1380	[73]
-	3	20	≈2 × 10^−3^ (*V_g_* of 20 V)	2.22	656	[74]
-	2.2	13.2	10^−2^ (*V_g_* of 13 V)	3.67	/	[76]
Junction gate (MIM)	2.2	14.1	10^−3^ (*V_g_* of 7 V)	3.16	/	[75]
Junction gate (PNJ)	1.78	19.35	≈8 × 10^−4^ (*V_g_* of 10 V)	3.84	600	[72]
Hybrid gate	1.8 (Ohmic + Schottky)	/	2 × 10^−2^ (*V_g_* of 6 V)	1.21	1090	[80]
-	1.7	/	4 × 10^−4^ (*V_g_* of 5 V)	30.1	650	[81]
-	1.2	/	/	3.3	1315	[84]

## Data Availability

No new data were created or analyzed in this study. Data sharing is not applicable to this article.
